# Clinical phenotype, *NOD2* genotypes, and treatment observations in Yao syndrome: a retrospective case series

**DOI:** 10.3389/fimmu.2024.1304792

**Published:** 2024-10-04

**Authors:** Katrina A. Williamson, Matthew J. Samec, Jenny A. Patel, Amir B. Orandi, Benjamin Wang, Cynthia S. Crowson, Edward V. Loftus, Afsaneh Alavi, Ann M. Moyer, John M. Davis

**Affiliations:** ^1^ Division of Rheumatology, Mayo Clinic, Rochester, MN, United States; ^2^ Department of Pediatric and Adolescent Medicine, Mayo Clinic, Rochester, MN, United States; ^3^ Division of Pediatric Rheumatology, Mayo Clinic, Rochester, MN, United States; ^4^ Division of Rheumatology, Mayo Clinic, Jacksonville, FL, United States; ^5^ Department of Quantitative Health Sciences, Mayo Clinic, Rochester, MN, United States; ^6^ Division of Gastroenterology and Hepatology, Mayo Clinic, Rochester, MN, United States; ^7^ Department of Dermatology, Mayo Clinic, Rochester, MN, United States; ^8^ Department of Laboratory Medicine and Pathology, Mayo Clinic, Rochester, MN, United States

**Keywords:** autoinflammatory syndromes, periodic fever syndromes, *NOD2*, Yao syndrome, case series design

## Abstract

**Objective:**

The aim of this study was to characterize the phenotype and genotype of patients with Yao syndrome (YAOS), with focus on comparing to prior cohorts, identifying novel features, and describing treatment observations.

**Methods:**

A retrospective medical records review of patients with YAOS seen at Mayo Clinic was conducted to characterize clinical features, *NOD2* genotypes, and therapeutic trials and responses.

**Results:**

Twenty-two patients diagnosed with YAOS were included. Eighteen patients (81.8%) were female and twenty (90.9%) were White. Mean age at symptom onset was 24.0 ± 14.8 years. Common clinical manifestations included fever (81.8% of patients), rash (95.5%), chronic gastrointestinal symptoms (100%), arthralgia/arthritis (95.5%), and sicca symptoms (68.2%). *NOD2* genotypes as single variants included IVS8 + 158 in 14 patients (63.6%), R702W in 8 patients (36.4%), 1007fs in 4 (18.2%), and one patient had only a previously unreported rare variant. Eight patients (36.4%) had compound (two or more) *NOD2* variants. Potential comorbidities of YAOS observed in this cohort included gastrointestinal dysmotility, autonomic dysfunction, and mast cell activation-like symptoms. Glucocorticoid responsiveness was observed in 15 of 20 patients exposed (75%). Eleven patients (50.0%) received IL-1 inhibitor therapy, and one patient (4.5%) received IL-6 inhibitor therapy with adequate disease control.

**Conclusion:**

Our findings substantiate the occurrence of fevers, arthralgia/arthritis, rash, chronic gastrointestinal symptoms, and sicca-like symptoms described previously in patients with YAOS. Novel clinical features and one *NOD2* variant not previously described were identified. Glucocorticoids, biologic IL-1 inhibitors, and IL-6 receptor inhibitors appeared to be effective for treatment of patients with YAOS.

## Introduction

The understanding of autoinflammatory diseases (AIDs) has rapidly evolved over recent decades with advances in genomic technologies and accessibility of genetic testing. AIDs are characterized by periodic fevers and inflammatory symptoms in various organ systems. The autoantibodies and chronic, progressive features that are characteristic of autoimmune diseases are notably absent in AIDs (1). Unlike autoimmune diseases, in which the adaptive immune system is integral to the pathogenesis, AIDs arise primarily from dysfunction of the innate immune system that can be associated with one or more genetic defects (1–6).

The nucleotide-binding oligomerization domain containing 2 (*NOD2*) gene has been identified previously as one linked to AIDs. The encoded NOD2 protein serves as an immune sensor for bacteria by recognizing the pathogen-associated molecular pattern muramyl dipeptide, activating the nuclear factor-kappa-B signal transduction pathway that mediates defense against microorganisms, regulating the immune response, and maintaining homeostasis (7). Certain *NOD2* sequence variants are suspected to result in dysregulation of immune/inflammatory responses, and loss-of-function variants are associated with Crohn’s disease while gain-of-function variants are associated with Blau syndrome (8–12).

More recently, specific *NOD2* variants have been associated with Yao syndrome (YAOS), previously known as *NOD2*-associated autoinflammatory disease, a polygenic AID with characteristic features of episodic fever, dermatitis, arthralgia/arthritis, pleuritis, gastrointestinal symptoms, distal extremity swelling, and sicca-like symptoms (13–17). Unlike Blau syndrome and monogenic AIDs, symptom development in YAOS frequently occurs in early to middle adulthood, though symptoms may develop earlier than this (10, 13, 14, 17–19).

Yao et al. first described the clinical phenotype of YAOS associated with *NOD2* gene variants in a published case series in 2011 (13). Seven patients were identified with manifestations of periodic fever, dermatitis, non-erosive polyarthritis, and serositis without evidence of other systemic autoimmune diseases or other AIDs (13). The patients all had *NOD2* variant IVS8 + 158 (also known as c.2798 + 158C>T, based on the NM_022162.3 transcript) with four additional patients also having the R702W variant (also known as c.2104C>T, p.Arg702Trp) (13). These patients appeared phenotypically distinct from pediatric Blau syndrome and Crohn’s disease and lacked granulomatous inflammation seen in these other NOD2-associated conditions. Subsequent articles have since described the expansion of clinical features and associated *NOD2* variants in YAOS (14–17, 19). However, most of what is known about YAOS has been reported by a single group.

Herein, we describe a case series of patients with YAOS seen at Mayo Clinic with the aim of corroborating prior descriptions and reporting possible novel phenotypes and genotypes of this increasingly recognized clinical syndrome. We further describe our clinical experience treating YAOS.

## Materials and methods

### Study design and patients

This was a retrospective case series of patients diagnosed clinically with YAOS by their primary rheumatologist at our institution. Mayo Data Explorer (MDE) is an institutional tool for exploration and retrieval of data in individual patients’ electronic health records (EHR). MDE was used to identify adult or pediatric patients with signed research authorization, at least one ICD-10 code for an autoinflammatory/periodic fever syndrome (M04.1, M04.8, M04.9, or D89.9), and detection of either “NOD2,” “CARD15,” or “Yao syndrome” as free text in patients’ clinical notes. Searches were last updated on April 1, 2024. A study investigator reviewed each patient’s EHR using a case report form to ascertain their eligibility. The study was reviewed and approved by the Mayo Foundation Institutional Review Board with waiver of informed consent.

Patients were required to meet a strict case definition to be included in the study. They were required to have a clinical diagnosis of YAOS by their treating rheumatologist at Mayo Clinic and to fulfill the published diagnostic criteria for YAOS (16). For the purpose of applying the exclusion criterion, high-titer ANA was defined as a titer of >1:320 by indirect immunofluorescence or value of >3.0 by enzyme immunoassay. Absence of listed exclusionary diagnoses was verified by reviewing physician diagnoses in the charts. Eligible patients were required to have documentation of genetic testing to exclude alternative AIDs. Genetic testing for each patient was ordered by the treating physician based clinical suspicion for an autoinflammatory disease. One patient underwent genetic testing only for *MEFV*, *TNFRSF1A*, and *NOD2* variants. All other patients underwent extensive genetic testing, using large commercially available panels or whole exome sequencing. Specific genetic screening tests completed in this cohort can be found in the **Supplementary Table 1 (S1)**.

**Figure 1:**
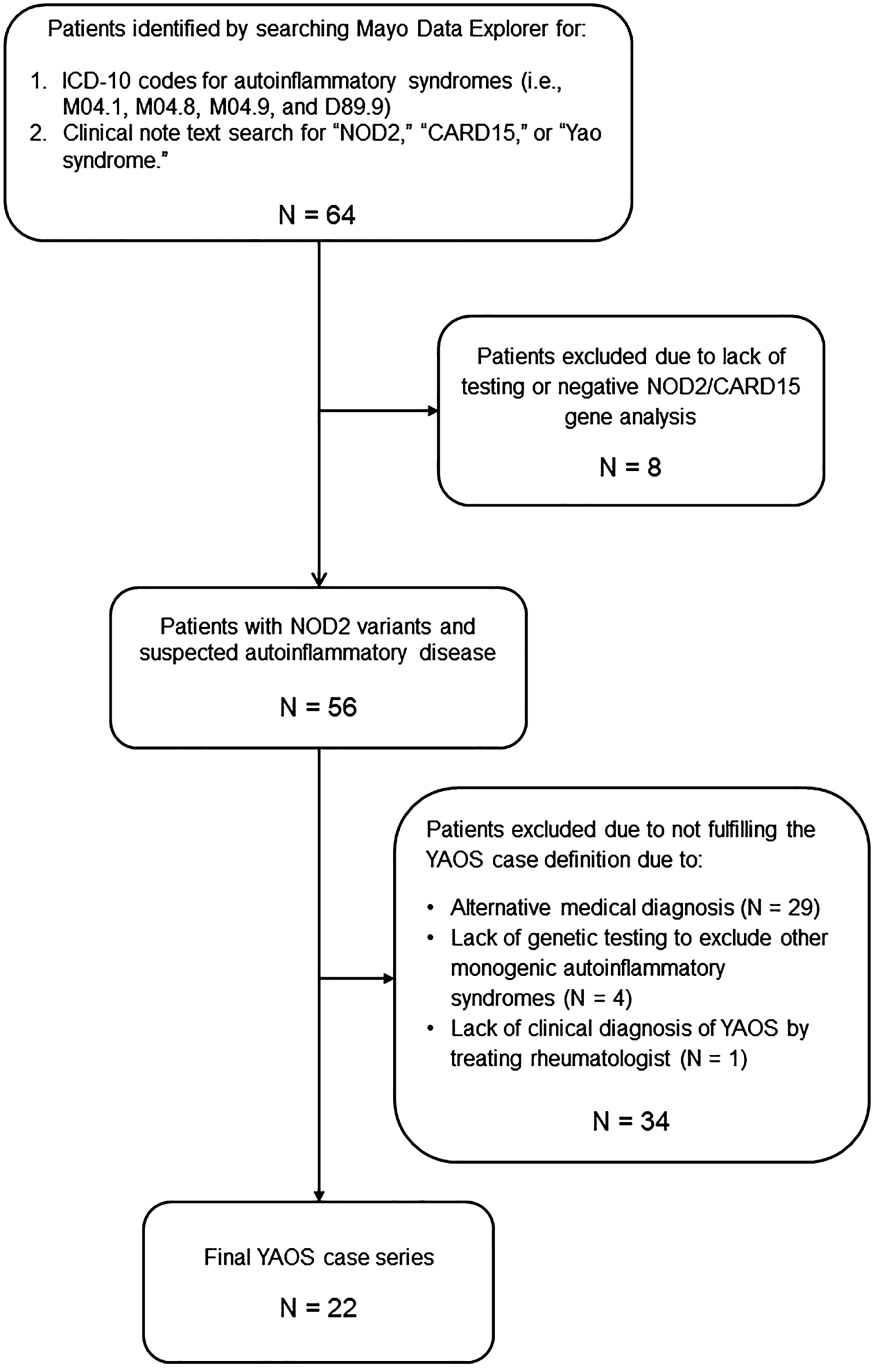
Assembly of the Mayo Clinic YAOS case series.

The assembly of the final YAOS case series is illustrated in **Figure 1**. The Mayo Data Explorer search identified 64 patients. Eight patients were excluded due to lack of documentation of *NOD2* test results or for testing negative for *NOD2* variants. Twenty-nine patients were excluded due to a diagnosis of an alternative medical condition to YAOS. Four excluded patients did not undergo genetic testing for periodic fever/autoinflammatory diseases beyond *NOD2*, and one patient lacked a clinical diagnosis of YAOS by the treating rheumatologist. The most common alternative diagnoses included undifferentiated or mixed autoinflammatory syndromes (11 cases), Blau Syndrome (3 cases), spondyloarthritis (3 cases), and alternative monogenic AIDs (3 cases). A total of 22 patients with YAOS were included in the final case series.

### Data collection

The electronic medical record was thoroughly reviewed for each patient, and data collection included demographics, associated comorbidities, YAOS characteristics (including clinical phenotype and *NOD2* genotype), laboratory and diagnostic test results, pathology results, clinical course, and treatments. Treatment trials and clinical response to medications were also abstracted.

### Statistical analysis

Continuous variables were expressed as mean and standard deviation and categorical variables as count and frequency/percentage.

## Results

### Demographics

The final case series consisted of 22 patients seen at Mayo Clinic and meeting diagnostic criteria for YAOS (**Table 1**). Among the 22 patients, 18 (82%) were female. There were 20 non-Hispanic White patients (90.9%), one Asian patient, and one Hispanic patient. The mean age at first clinical manifestation was 24.0 ± 14.8 years (range 2 – 59 years). The mean age of YAOS diagnosis was 36.1 ± 13.1 years (range 11 – 71 years). One patient was diagnosed by a pediatric rheumatologist at age 11; all other cases were adults (age ≥18 years) at diagnosis. Duration of symptoms prior to YAOS diagnosis was 11.6 ± 6.8 years. Eight patients (36%) had previously been diagnosed with an autoimmune disease, later revised to YAOS. Prior diagnoses included atypical adult-onset Still’s disease (N = 1), seronegative rheumatoid arthritis (N = 3), seronegative or undifferentiated inflammatory arthritis (N = 2), and undifferentiated autoimmune disorder (N = 2). Five patients (22.7%) reported a family history of suspected autoinflammatory disease. None of the included patients had a family history of confirmed monogenic AID, and none were known relatives.

**Table 1:** Demographics, clinical, and laboratory features of patients with YAOS diagnosed at Mayo Clinic.

Clinical Feature	Value^*^ (N = 22)
Demographics	
Female sex	18 (81.8)
White race	20 (90.9)
Clinical characteristics	
Age at first clinical symptom onset, mean ± SD (range), years	24.0 ± 14.8 (2 – 59)
Age at YAOS diagnosis, mean ± SD (range), years	36.1 ± 13.1 (11 – 71)
Disease (symptoms) duration prior to YAOS diagnosis, mean ± SD (range), years	11.6 ± 6.8 (0.9 – 25)
Prior autoimmune disease diagnosis	8 (36.4)
Family history	5 (22.7)
Clinical phenotype	
Fever	18 (81.8)
Periodic pattern	10/18 (55.5)
Sporadic pattern	6/18 (33.3)
Daily pattern	2/18 (11.1)
Fatigue	22 (100)
Night sweats	15 (68.2)
Weight loss	11 (50)
Lymphadenopathy	11 (50)
Skin manifestations	21 (95.5)
Erythematous patches and plaques	17/21 (81.0)
Facial erythema	14/21 (66.7)
Trunk involvement	13/21 (61.9)
Eyelid swelling	7/21 (33.3)
Skin biopsy	11 (52.4)
Spongiotic dermatitis	3/11 (27.3)
Perivascular lymphocytic inflammation	5/11 (45.5)
Other dermatitis†	3/11 (27.3)
Oral ulcerations	10 (45.5)
Arthralgia/arthritis	21 (95.5)
Upper and lower extremity involvement	19/21 (90.5)
Polyarticular (≥4 joints)	18/21 (85.7)
Small and large joint involvement	18/21 (85.7)
Inflammatory arthritis	8/21 (38.1)
Large joint involvement only	2/21 (9.5)
Lower extremity involvement only	2/21 (9.5)
Myalgia	16 (72.7)
Distal lower extremity swelling	17 (77.3)
Any Gastrointestinal Symptoms^‡^	22 (100)
Abdominal pain	21 (95.5)
Diarrhea	20 (90.9)
Nausea or vomiting	17 (77.3)
Abdominal bloating	17 (77.3)
Recurrent chest pain	13 (59.1)
Recurrent pleurisy	7/13 (53.8)
Dizziness	15 (68.2)
Orthostatic intolerance	10/15 (66.7)
Postural orthostatic tachycardia syndrome	4/15 (26.7)
Asthma	16 (66.7)
Headaches	17 (77.3)
Sicca symptoms	15 (68.2)
Nephrolithiasis	3 (13.6)
Laboratory features	
Leukocytosis	3 (13.6)
Anemia^#^	9 (40.9)
Elevated C-reactive protein	4 (18.2)
Elevated sedimentation rate	4 (18.2)

^*^Values represent number (%) unless otherwise specified.

^†^One biopsy demonstrated non-specific dermal inflammatory infiltrate alone, one biopsy (of facial papules) demonstrated pityrosporum infection, and one demonstrated skin ulceration with acute folliculitis with focal gram positive cocci with background of superficial and deep diffuse mixed inflammation including focal small aggregates of neutrophils.

^‡^Gastrointestinal symptoms included abdominal pain, diarrhea, abdominal bloating, and nausea and/or vomiting.

^#^Three of the nine patients had iron deficiency anemia.

### Clinical features

#### Flare patterns

There was significant heterogeneity in the pattern of flares in this cohort. In particular, there was significant variability in duration of flare symptoms and interval between episodes. Fever and skin manifestations were the most common clinical symptoms to occur periodically (in discrete episodes ranging from a few days to several months followed by symptom-free intervals). Other symptoms, such as gastrointestinal symptoms, musculoskeletal symptoms, and fatigue were frequently persistent, often occurring daily. Rash and fever symptoms were noted to flare concordantly at times in some individuals and independently in others. Vaccination and infection were the most commonly identifiable triggers for symptom flares.

#### Constitutional

Fever was present in 18 (81.8%) patients. Fever patterns were characterized as periodic (occurring in discrete episodes followed by periods of remission without fever) in 10 patients (55.5%), sporadic (irregular occurrence including in single occurrences or multiple occurrences per week) in six patients (33.3%), and daily in two patients (11.1%). The duration of febrile episodes and intervals between episodes were highly variably between patients, even among individual patients over time. Fatigue was reported in all patients. Night sweats, unintentional weight loss, and lymphadenopathy were also common. Lymphadenopathy involving the cervical lymph nodes was most common, occurring in eight of the 11 patients with lymphadenopathy (72.7%). Other regions of lymphadenopathy included inguinal (2 patients), axillary (3 patients), and supraclavicular (1 patient). Seven of the 11 cases with lymphadenopathy had enlarged lymph nodes confirmed on imaging (computed tomography, magnetic resonance imaging, or ultrasound) with the remainder having palpable lymph nodes noted by the patient or provider during symptom flares.

#### Cutaneous

**Figure 2:**
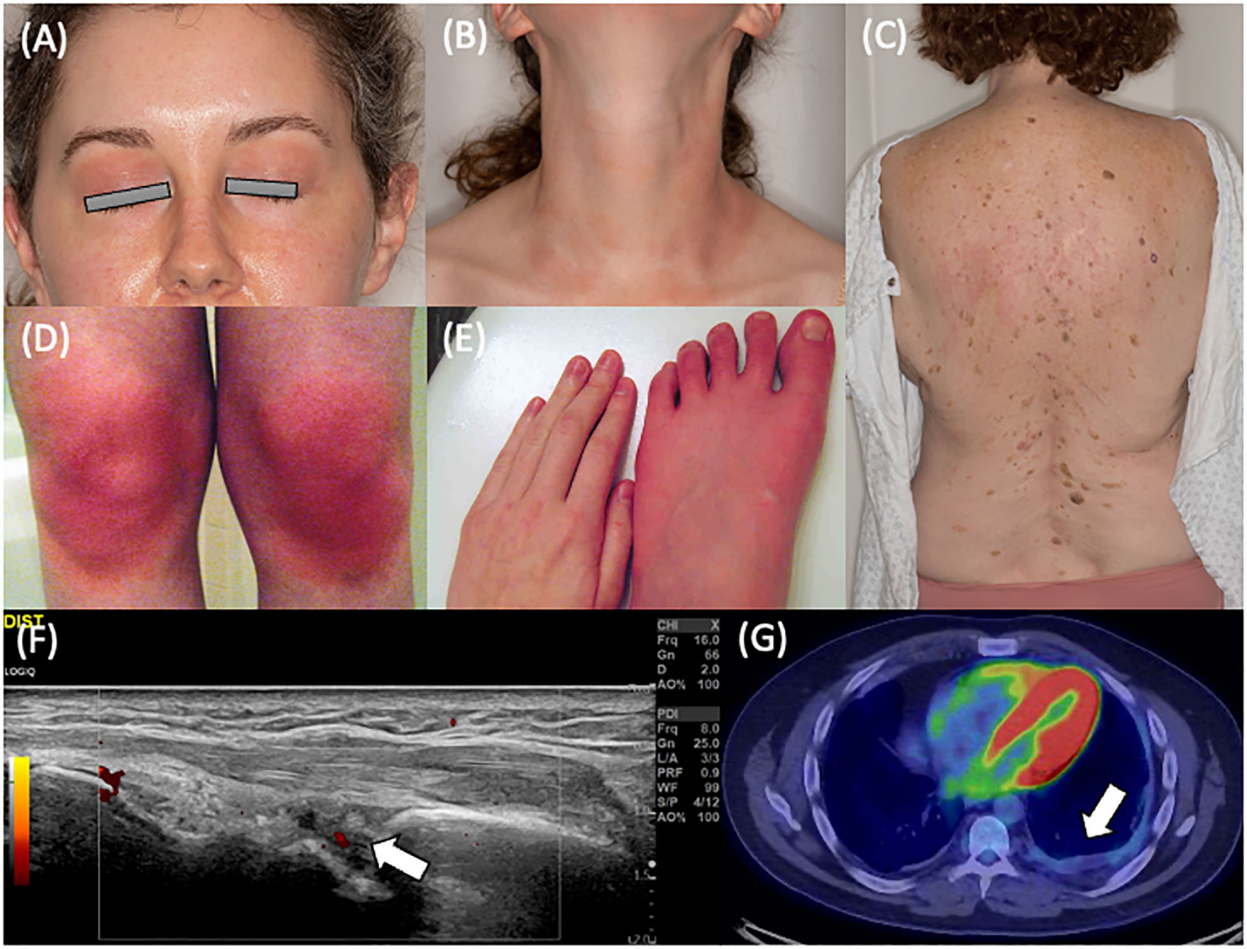
Clinical features of YAOS in the Mayo Clinic cohort. **(A)** Eyelid redness and swelling and **(B)** erythematous patches and plaques on the anterior neck of a patient with *NOD2* IVS8 + 158 and R702W variants. **(C)** Erythematous maculopapular eruption on the back of a patient with *NOD2* IVS8 + 158 and R702W variants. Dermatopathology revealed focal follicular mucinosis and mild perivascular lymphocytic inflammation. **(D)** Cutaneous flushing overlying the bilateral knees and **(E)** distal extremity swelling and erythema in a patient with *NOD2* IVS8 + 158 and R702W variants. **(F)** Musculoskeletal ultrasonography of the dorsal radiocarpal joint in longitudinal scan, demonstrating grayscale and power Doppler synovitis in a patient with *NOD2* IVS8 + 158 variant. **(G)** Positron emission tomography showing left basilar pleural thickening with faint fluorodeoxyglucose avidity in a patient with restrictive lung disease in a patient with two copies of NOD2 IVS8 + 158, R702W, and 1007fs variants.

Twenty-one patients (95.5%) had cutaneous manifestations with one or more of recurring erythema/flushing, discrete rash, or eyelid swelling. Facial erythema was commonly noted with malar rash specifically in eight patients. Eyelid swelling with or without erythema (**Figure 2A**) was noted in seven patients (33.3% of patients with cutaneous manifestations). Erythematous patches and plaques as previously described in YAOS were noted in 17 patients (81.0% of those with skin manifestations) and could be pruritic or non-pruritic. Rash affected various regions, including the face, neck (**Figure 2B**), extremities, and trunk (**Figure 2C**), with the trunk involved in 13 patients. One patient had episodic transient erythematous flushing overlying her knees (**Figure 2D**) and erythema and swelling of her feet (**Figure 2E**). Other descriptions of rash present in the cohort included livedo reticularis (N = 4), papular (N = 5), maculopapular (N = 2), and petechial or ecchymotic rash (N = 3). Eleven patients underwent skin biopsy with dermatopathology from five biopsies demonstrating perivascular lymphocytic inflammation and three demonstrating spongiotic dermatitis. Clinical rash appearance did not correlate to specific histopathologic findings aside from biopsies completed of petechial/ecchymotic lesions in two patients both demonstrating perivascular lymphocytic inflammation without vasculitis. Ten patients (45.5%) experienced oral ulcerations.

#### Musculoskeletal

Joint symptoms were present in 21 patients (95.5%). Of these, eight patients (38.1%) had inflammatory arthritis (**Figure 2F**). Joint involvement was most commonly polyarticular (85.7% of cases with joint manifestations) with both small and large joint involvement (85.7%) and affecting both upper and lower extremity joints (90.5%). Involvement was otherwise oligoarticular in the lower extremities, including the knees in these two cases. Joint radiographs were performed in 16 of the 21 patients with no erosive changes identified. Myalgia was present in 16 patients (72.7%), and distal lower extremity swelling was present in 17 patients (77.3%). Distal extremity swelling most often occurred bilaterally (82.4%) and could occur with or without erythema in the affected site. Sites of extremity swelling included the ankles, feet, and hands.

#### Cardiopulmonary

Dizziness (non-vertiginous) was common, occurring in 15 patients (68.2%). There was a spectrum of pathology underlying dizziness that included orthostatic intolerance (defined as the association of lightheadedness, dizziness, or syncope occurring with prolonged standing or upright position (20)), postural orthostatic tachycardia syndrome (POTS), and orthostatic hypotension. Orthostatic intolerance was present in ten patients (two-thirds of those with dizziness). Two patients (13.3%) had orthostatic hypotension noted. Postural orthostatic tachycardia syndrome (POTS) was diagnosed by tilt table examination in four patients (26.7% of those with dizziness). Recurrent chest pain was noted in 13 patients (59.1%). Seven of these patients described pleuritic chest pain and were diagnosed with recurrent pleurisy. One patient had objectively abnormal testing demonstrating pleural effusion and pleural thickening. Four patients had a history of pericarditis. Asthma was diagnosed in 16 patients (72.7%) with no cases of interstitial lung disease. Two patients had recurrent bronchitis. There was one case of restrictive lung disease secondary to pleural thickening (**Figure 2G**).

#### Gastrointestinal

Gastrointestinal symptoms (one of more of abdominal pain, bloating, chronic nausea, or diarrhea) occurred in all patients. Recurrent abdominal pain was noted in 21 patients (95.5%) and diarrhea in 20 patients (90.9%), with five patients endorsing at least one occasion of bloody stools (in the absence of inflammatory bowel disease [IBD]). Abdominal bloating and nausea were also frequently reported (77.3% for each). Seventeen patients underwent colonoscopy, and 8 patients underwent ileal biopsy, with findings considered to be normal in all cases. Of note, one patient was reported to have a non-specific isolated lymphoid aggregate. No patients had any histopathologic evidence of Crohn’s disease or ulcerative colitis. Two patients were diagnosed with collagenous colitis and one with non-specific mild focal active colitis by colon biopsy. Fifteen patients underwent upper endoscopy, revealing eosinophilic esophagitis in one patient, celiac disease in two patients, chronic gastritis in four patients (one eosinophilic gastritis and one collagenous gastritis), and duodenitis in two patients. Seven patients were diagnosed with gastrointestinal dysmotility (with cases of gastric, small intestine, and colon dysmotility). Three of these cases were confirmed with abnormal motility studies. There were five patients diagnosed with pelvic floor dysfunction, eight patients with irritable bowel syndrome, and one patient with biliary dyskinesia. One patient had recurring anal fissures. Two patients were diagnosed with pancreatic insufficiency and there was one case of recurrent pancreatitis. There was one patient with abnormal liver function tests identified during periods of clinical flare without an alternative etiology for liver dysfunction.

#### Neurologic

Recurring headaches were noted in 17 patients (77.3%). No patients were found to have signs of autonomic failure. One patient was diagnosed externally with dysautonomia but had normal tilt table and sweat testing at our institution. One patient was found to have a small fiber neuropathy, with abnormal findings on both autonomic testing and skin biopsy, which showed reduced epidermal nerve fiber density. This patient was also diagnosed with a movement disorder manifesting with myoclonus.

#### Sicca-like symptoms and other manifestations

Fifteen patients (68.2%) experienced sicca symptoms. None were diagnosed with inflammatory eye disease, although four patients described recurrent flares of eye redness. Three patients (13.6%) had history of nephrolithiasis. One patient was diagnosed and concurrently treated for mast cell activation disorder based on elevated an elevated tryptase and urinary N-methylhistamine levels, and another patient had a clinical diagnosis by an allergist at another institution with partial response to treatment with ketotifen. An additional ten patients experienced repeated flushing episodes that raised concern for mast cell activation disorder but had normal blood tryptase levels.

#### Pediatric onset

The earliest onset of symptoms in this cohort was age 2 and noted to be recurrent fevers in the absence of identifiable infection. Ten patients (45.5%) were less than 18 years of age at symptom onset, with earliest symptoms including one or more of recurring fevers, rashes, arthralgia, and gastrointestinal symptoms. Only one patient was diagnosed with YAOS earlier than age 18, specifically at age 11.

### Laboratory findings

Patients underwent extensive rheumatic disease evaluations and were commonly evaluated for ANA-associated diseases, rheumatoid arthritis, ANCA-associated vasculitis, and HLA-B27-associated spondyloarthritis based on clinical symptoms. Full details of these serological test results are available in the **Supplementary Table 2**. Seven patients were found to have a positive ANA (five patients ANA-positive, one with isolated anti-RNP antibody, and another with isolated SSB antibody). ANA titers were predominantly low, with immunofluorescence titers ranging from 1:40 to 1:320. Only one ANA-positive patient was found to have more specific autoantibodies (anti-SSB), and none demonstrated positive anti-double-stranded DNA antibodies. Two patients in total were positive for anti-SSB antibodies. Neither patient was positive for anti-SSA antibodies and neither patient diagnosed with Sjogren’s syndrome. Rheumatoid factor and anti-CCP were checked in 21 and 18 patients respectively and negative in all cases. Of 14 patients tested for ANCA, one was positive for anti-proteinase-3 antibody with negative c-ANCA and p-ANCA, and all others had negative results. Eleven patients were tested and negative for HLA-B27.

Laboratory data of the patients with YAOS demonstrated anemia present in nine cases (40.9%), although this was related to iron deficiency in three patients. Leukocytosis (episodic) was present in three cases (13.6%). Elevation of either the C-reactive protein (CRP) or sedimentation rate (ESR) was present in six cases (27.3%), including two with both elevated CRP and ESR and two with elevated ESR alone.

### NOD2 genotype frequencies

*NOD2* genotypes are described in **Table 2**. Among the 22 patients, 14 (63.6%) had the IVS8 + 158 (c.2798 + 158C>T) variant, 8 patients (36.4%) had R702W (c.2104C>T, p.Arg702Trp), and 4 had 1007fs (c.3019dup, p.Leu1007Profs*2). Seven patients (31.8%) had only IVS8 + 158, and two patients (9.1%) had only R702W. Eight patients (36.4%) had compound *NOD2* variants, most commonly IVS8 + 158 plus either R702W or 1007fs. Two patients had rare *NOD2* variants (c.931C>T, p.R311W and c.2107C>T, p.Arg703Cys in one patient and c.2863G>A, p.Val955lle in another). Three patients had non-diagnostic (variants of unknown significance not known to be pathogenic) heterozygous variants in other immune genes, including NLRP1, LPIN2, RNASEH2B, TNFRSF6B, TREX1, VPS13B, and AIRE (**Table 2**).

**Table 2:** *NOD2* genotypes in the Mayo Clinic YAOS case series compared to a previous study.

*NOD2* Variants	Mayo Clinic (N = 22)	Yao et al.^16^ (N=54)
Single Variants		
c.2798 + 158C>T, p.? (IVS8 + 158C>T)^*^	14 (63.6)	46 (85)
c.2104C>T, p.Arg702Trp (R702W)^†^	8 (36.4)	15 (28)
c.3019dup, p.Leu1007Profs*2 (1007fs)	4 (18.2)	2 (4)
c.2722G>C, p.Gly908Arg (G908R)	1 (4.5)	1 (2)
c.931C>T, p.Arg311Trp (R311W)	1 (4.5)	0 (0)
c.2107C>T, p.Arg703Cys (R703C)	1 (4.5)	1 (2)
c.2863G>A p.Val955lle (V995I)	1 (4.5)	0 (0)
Compound Variants		
c.2798 + 158C>T (IVS8 + 158C>T) + c.2104C>T, p.Arg702Trp (R702W)^‡^	5 (27.3)	14 (26)
c.2798 + 158C>T (IVS8 + 158C>T) + c.3019dup, p.Leu1007Profs*2 (1007fs)	1 (4.5)	1 (2)
c.2798 + 158C>T (IVS8 + 158C>T) + c.2104C>T, p.Arg702Trp (R702W) + c.3019dup, p.Leu1007Profs*2 (1007fs)^@^	1 (4.5)	1 (2)
c.931C>T, p.Arg311Trp (R311W) + c.2107C>T, p.Arg703Cys (R703C)^#^	1 (4.5)	0 (0)

Variants are defined according to HGVS nomenclature with common names in parentheses. Values are number (%).

^*^Seven patients had only the IVS8 + 158C>T variant.

^†^Two patients had only the R702W variant. One of these patients was identified to have a heterozygous variant of unknown significance in the NLRP1 gene, c.2311G>A, p.Glu771Lys.

^‡^One patient had heterozygous LPIN2 c.2656C>G, p.Pro886Ala; RNASEH2B c.787A>G, p.Thr263Ala; and TNFRSF6B c.302A>C, p.Glu101Ala. One patient was identified to also have an AIRE gene pathogenic variant c.967_979del, p.Leu323Serfs*51. One patient had a heterozygous variant of unknown significance in the IL10RA gene, c.884C>T, p.Pro295Leu and heterozygous variant of unknown significance VPS13B c.7955A>C, p.Asn2652Thr.

^@^Patient had two copies of IVS8 + 158C>T in addition to other listed variants.

^#^Patient had only two NOD2 variants of unknown significance, with only c.2107C>T, p.Arg703Cys (R703C) previously described in YAOS^16^. This patient also was heterozygous (carrier status rather than disease status) for a pathogenic mutation in TREX1 c.341G>A, p.Arg114His.

### Treatment experience

The patients were treated with a variety of medications over their disease course (**Table 3**). Therapies included glucocorticoids, colchicine, conventional synthetic disease-modifying antirheumatic drugs (DMARDs), and biologic DMARDs. Non-steroidal anti-inflammatory drugs were used for symptom management in a small number of patients but were generally ineffective. Twenty patients (90.9%) were treated with glucocorticoids with benefit noted in 15 (75%). Colchicine was tried in eight cases, with one patient having reduction in oral ulcers, one treated for pericarditis, and one patient who improved subjectively but ultimately transitioned to anakinra for continued disease activity. Three patients were maintained on conventional DMARD therapy alone. One patient was on methotrexate monotherapy, one on methotrexate and leflunomide, and another on methotrexate and sulfasalazine. Of these three patients, only the patient on methotrexate monotherapy was reported to have good disease control on this therapy. Fourteen patients (63.6%) trialed sulfasalazine. Five patients experienced allergic reaction or intolerable side effects. Only one of the remaining patients experienced symptomatic improvement in musculoskeletal pain, headache, and diarrhea symptoms, and the remaining patients experienced insufficient benefit. Patients were trialed on a variety of biologics, including tumor necrosis factor (TNF) inhibitors, IL-1 inhibitors, and IL-6 receptor inhibitors. There was also infrequent use of rituximab, abatacept, and secukinumab. Two patients (9.1%) were observed off any immunosuppressive therapy. Thirteen patients (59.1%) achieved adequate disease control on therapy, including eleven patients who were maintained on IL-1 inhibitor therapy alone or combined with conventional synthetic DMARD therapy and one patient treated long-term with IL-6 receptor inhibitors.

**Table 3:** Summary of therapeutic trials among the patients with YAOS at Mayo Clinic.

Case Number	Glucocorticoid responsive	Failed DMARDs	Beneficial DMARDs	Most recent Drug Regimen
Case 1	Yes	Hydroxychloroquine, sulfasalazine, methotrexate monotherapy, adalimumab, infliximab, IVIG, anakinra (intolerable side effects and injection site reactions), canakinumab (intolerable side effects), tocilizumab (recurring symptoms two weeks after infusions, infectious complications)	Sarilumab (used in combination with methotrexate, which benefited arthritis). Loss of efficacy noted after about 34 months. Colchicine utilized for recurring aphthous ulcers.	Etanercept (efficacy not yet assessed)
Case 2	Yes, maintained on chronic prednisone	Hydroxychloroquine, etanercept, sulfasalazine (intolerable side effects, possible hypersensitivity), methotrexate, IVIG (anaphylactic reaction), colchicine, mycophenolate mofetil, azathioprine, abatacept, anakinra	Canakinumab (early recurrence on monthly injections), sarilumab (unable to tolerate effective dose due to neutropenia)	Chronic prednisone 10mg daily, canakinumab 150mg every two weeks
Case 3	Yes (pleurisy/pleuritis)	Colchicine	Methotrexate	Methotrexate
Case 4	Yes	Hydroxychloroquine, sulfasalazine (side effects)		Off therapy (without indication for immunosuppression)
Case 5	Yes	Hydroxychloroquine, sulfasalazine (side effects), methotrexate, canakinumab	Anakinra (unable to get insurance approval for long-term use) Rilonacept	Rilonacept
Case 6	Yes	Hydroxychloroquine, methotrexate, adalimumab (generalized swelling reaction), tocilizumab (side effects, severe abdominal pain), anakinra (waning efficacy), rituximab (side effects nausea)	Colchicine (pericarditis), mycophenolate (limited course for serositis), canakinumab (effective, side effects including fatigue and hypersomnolence)	Canakinumab 150mg every four weeks
Case 7	Unclear	Hydroxychloroquine monotherapy, sulfasalazine, methotrexate	Hydroxychloroquine with anakinra	Hydroxychloroquine, anakinra 200mg daily
Case 8	No	Sulfasalazine, canakinumab	Sarilumab	Sarilumab 200mg every 14 days
Case 9	No	Sulfasalazine, colchicine	Canakinumab	Canakinumab 150mg every 8 weeks
Case 10	Yes	Hydroxychloroquine, methotrexate with hydroxychloroquine	Assessment on most recent therapy unavailable	Tocilizumab 162mg every 14 days, methotrexate 20mg weekly
Case 11	No	Hydroxychloroquine, methotrexate (gastrointestinal side effects), abatacept, anakinra (injection site reactions)	Anakinra	Anakinra 100mg daily
Case 12	Not used	Sulfasalazine monotherapy, canakinumab 150mg every four weeks and sulfasalazine, methotrexate (worsening), tocilizumab	Canakinumab 300mg every four weeks	Canakinumab 300mg every four weeks
Case 13	Not used	Sulfasalazine (side effects), colchicine		Observed off therapy
Case 14	Yes	Hydroxychloroquine, adalimumab (anaphylactic reaction), methotrexate with leflunomide, anakinra (hives)		Leflunomide 20mg daily with methotrexate 20mg weekly (while awaiting allergy evaluation for medication allergic reactions)
Case 15	Yes	Hydroxychloroquine, sulfasalazine, methotrexate, mycophenolate mofetil, azathioprine, abatacept, etanercept, tocilizumab	Certolizumab (duration of therapy approximately 9 years, therapy transitioned due to increasing rate of break-through flares), rituximab (therapy transitioned due to frequent infections) anakinra	Anakinra 100mg daily
Case 16	Yes, maintained on chronic prednisone		Canakinumab (Improvement in rash and fevers, but incomplete response of MSK and GI symptoms), prednisone	Canakinumab 300mg every 4 weeks, sulfasalazine 1000mg daily (add-on therapy), prednisone 7.5mg daily
Case 17	Yes	Plaquenil, azathioprine (side effects)	Anakinra 100mg daily 1(partial response of joints, Gi tract, fatigue, but intolerable palpitations)	Declined starting new therapy (etanercept)
Case 18	Yes	Sulfasalazine	Hydroxychloroquine (partial response, improvement in myalgia/arthralgia), methotrexate (improvement in arthralgia), etanercept (partial response, joint pain and swelling improved), canakinumab 150mg every four weeks (good global response)	Canakinumab 150mg every four weeks, hydroxychloroquine, and methotrexate
Case 19	Yes	Colchicine	Anakinra 100mg daily	Anakinra 100mg daily
Case 20	Yes	Etanercept	Colchicine (symptomatic improvement), dapsone (improvement in rash symptoms)	Anakinra 100mg daily
Case 21	Yes	Methotrexate plus sulfasalazine and naproxen/esomeprazole (Vimolo) combination therapy		Methotrexate plus sulfasalazine and naproxen/esomeprazole (Vimolo) combination therapy
Case 22	Unclear	Sulfasalazine (not tolerated), IVIG (trialed for possible autoimmune encephalopathy, not tolerated)	Canakinumab 150mg every four weeks	Canakinumab 150mg every four weeks

## Discussion

The findings of this study contribute to the limited literature on *NOD2*-associated disease. We reaffirm the basic definition of YAOS (OMIM Entry #617321) as a syndrome of episodic fever, rash, arthralgia/arthritis, chronic gastrointestinal symptoms, and sicca-like symptoms and describe novel clinical features identified in a sizeable cohort along with a previously unreported *NOD2* variant (13, 14, 16, 17, 21). We further describe treatment observations, which may be helpful in focusing future studies on management of YAOS.

Comparison of our data to published YAOS cohorts reveals striking similarities for most disease characteristics (14, 16, 17, 19, 21). The female predominance of our cohort is like previous studies (14, 16, 17, 19), and the non-Hispanic White racial predilection is similar to previous North American cohorts (14, 16, 17). Our study highlights the frequent onset of symptoms of YAOS well into adulthood, but demonstrated wide variability ranging from symptom onset in the first years of life to onset in middle-late adulthood. Most patients suffered for many years prior to diagnosis. Similar to previous cohorts, the signs and symptoms of YAOS may be periodic, with a definable flare and remission pattern, sporadic, having an irregular pattern without definable episodes or periods of remission, or chronic, often daily symptoms, with periodic flares (14, 19).

Like prior studies, rashes frequently consisted of erythematous patches and plaques with dermatopathology revealing spongiotic dermatitis or perivascular lymphocytic inflammation (13, 14, 18). The absence in this cohort of cutaneous manifestations seen in the other *NOD2*-associated diseases, Blau syndrome and Crohn’s disease (e.g., erythema nodosum or pyoderma gangrenosum) is consistent with previous studies, although sparse numbers with granulomatous dermatitis have been reported previously (13, 14, 22). The Chinese case series of three patients with YAOS differed notably in the absence of rash (19). Arthralgia/arthritis was predominantly non-erosive and polyarticular, consistent with prior descriptions (13, 14, 17, 18). Characteristic distal extremity swelling with was observed as previously described (16, 19, 23). Evaluations for chronic gastrointestinal symptoms were universally negative for IBD (i.e., Crohn’s disease or ulcerative colitis). Laboratory abnormalities including leukocytosis and elevated acute phase reactants were comparable to published findings.

Novel observations in our cohort included a few cases with livedo and petechial-type rashes, which have not been described in YAOS. In addition to facial erythema that has previously been described, we further noted episodic facial flushing in twelve patients (54.5%) that resembled mast cell activation disorder. Two of these patients were ultimately diagnosed with mast cell activation disorder in addition to YAOS although only one of these patients had elevations of trypase and urinary N-methylhistamine levels documented in available medical records. Chest pain has been described in approximately 13 to 36 percent of YAOS patients (13, 14, 17, 19). Recurrent chest pain was more prevalent in this cohort, occurring in 59.1% of patients with half of these attributed to pleurisy/pleuritis. Pericarditis was less frequent and seen in just four patients. Recurrent dizziness was a novel symptom present in over two-thirds of this cohort and has not been previously detailed in YAOS. There was a spectrum of orthostatic intolerance to orthostatic hypotension and postural orthostatic tachycardia syndrome underlying dizziness, which has also not been previously described. Autonomic failure was not seen. Additionally, gastrointestinal dysmotility was present in seven cases and not previously described in YAOS. Several additional gastrointestinal processes such as presence of pelvic floor dysfunction, pancreatic and biliary disease were also noted and novel findings in this YAOS cohort. It is uncertain if these clinical features of mast cell activation, dizziness and orthostatic symptoms, and dysmotility conditions are causally related to YAOS or NOD2, comorbidities, or unrelated conditions.

YAOS is considered a multifactorial autoinflammatory disorder to which susceptibility is conferred by specific *NOD2* variants (10). It is proposed to be a genetically transitional disease, whereby an associated low penetrance *NOD2* variant is necessary but insufficient to cause the disease, and disease expression is mediated through interaction with other genetic or environmental factors (24). *NOD2* IVS8 + 158 (c.2798 + 158C>T) and R702W (c.2104C>T, p.Arg702Trp) have consistently been the most prevalent single variants in patients with YAOS, whereas 1007fs (c.3019dup, p.Leu1007Profs*2) and other low frequency or rare variants have been observed in small numbers of patients (13, 14, 17–19). The R702W and 1007fs variants were more prevalent in this cohort compared to the first large cohort of patients with YAOS described by Yao et al. (**Table 2**) (16). V955I (c.2863G>A, p.Val955Ile) has recently been identified as another YAOS susceptibility variant (25). One novel *NOD2* variant, R311W (c.931C>T, p.Arg311Trp) was also identified in this cohort. Over half of our cohort had compound variants, which is higher than the first large cohort study (14) but similar to the frequency in the most recent case series by Yao et al. (17). Association between specific *NOD2* genotypes and severity of disease or refractoriness to treatment were not clearly observed. A few patients were observed to have variants of unknown significance in other immune-related genes, including NLRP1 and AIRE. Patients with pathogenic, disease-causing mutations in other immune-related genes known to cause autoinflammatory disease were excluded.

Patients received numerous medications over the course of symptoms even prior to the diagnosis of YAOS. Most patients responded favorably to moderate-to-high-dose prednisone as previously reported. Colchicine, non-steroidal anti-inflammatories, and hydroxychloroquine have generally been ineffective (13, 14, 16, 17). Although noted to be effective in previous studies (16), sulfasalazine was poorly tolerated by patients in this cohort and was generally inadequate for disease management with just one patient noting some symptomatic improvement in pain and gastrointestinal symptoms that persisted despite canakinumab therapy. Most patients were ultimately maintained on IL-1 targeted therapy with a few on IL-6 targeted therapy. Similar success with IL-1 and IL-6 blockade has previously been described (13, 16, 17, 26, 27). Treatment was frequently complicated by side effects or intolerance to therapies or waning effectiveness.

## Limitations

The primary limitations of this study relate to its retrospective nature, which allowed for a larger cohort but resulted in variable duration of follow-up, and data availability (e.g., patient symptoms, diagnostics, and treatment history and efficacy). The approach to identify YAOS patients, which required documentation pertaining to *NOD2* or YAOS and diagnosis code for autoinflammatory syndromes along with a clinical diagnosis of YAOS by a patient’s rheumatology provider, may have missed some cases of YAOS but provided for a more thorough documentation of autoinflammatory signs and symptoms and minimized risks of misclassification. The limitations of the study design precluded correlation of NOD2 genotype with clinical features and treatment responses.

## Conclusion

The findings of this retrospective case series corroborate the previously reported clinical phenotypes and genotypes of patients with YAOS and expand upon these with new observations. It is uncertain whether gastrointestinal dysmotility, dysautonomia (e.g., POTS, orthostasis), and mast cell activation disorder are comorbidities or mechanistically related to NOD2 variants or the diagnosis of YAOS. A novel *NOD2* variant associated with YAOS is also described. This study contributes to the limited evidence on therapeutic options for YAOS, for which glucocorticoids, IL-1 inhibitors, and IL-6 receptor inhibitors appear to be effective. Further research is needed to identify the most efficacious dosing of these therapies and adjunctive therapies.

## Data Availability

The original contributions presented in the study are included in the article/**Supplementary Material**. Further inquiries can be directed to the corresponding author.

## Supplementary material

Supplementary Table 1 Genetic Screening Methods for Autoinflammatory Disease.

Supplementary Table 2 Laboratory results in the Mayo Clinic YAOS cohort. ^*^Results show the number of patients with positive tests/number of patients (%) tested. ^†^Immunofluorescence testing ranging from less than 1:40 to 1:320. Only one patient positive for ANA by immunofluorescence testing was found to have more specific autoantibodies (anti-SSB) and none demonstrated positive anti-double-stranded DNA antibodies. The second patient positive for anti-SSB had a negative ANA by immunofluorescence. ^‡^One patient was ANA negative. *^#^* One patient was PR3 positive with negative c-ANCA and p-ANCA immunofluorescence testing.

## References

[1] Rubartelli A. Autoinflammatory diseases. Immunol Lett. (2014) 161:226–30. doi: 10.1016/j.imlet.2013.12.013 24452074

[2] McDermott MF Aksentijevich I Galon J McDermott EM Ogunkolade BW Centola M. Germline mutations in the extracellular domains of the 55 kDa TNF receptor, TNFR1, define a family of dominantly inherited autoinflammatory syndromes. Cell. (1999) 97:133–44. doi: 10.1016/S0092-8674(00)80721-7 10199409

[3] Kastner DL Aksentijevich I Goldbach-Mansky R. Autoinflammatory disease reloaded: a clinical perspective. Cell. (2010) 140:784–90. doi: 10.1016/j.cell.2010.03.002 PMC3541025 20303869

[4] Sag E Bilginer Y Ozen S. Autoinflammatory diseases with periodic fevers. Curr Rheumatol Rep. (2017) 19:41. doi: 10.1007/s11926-017-0670-8 28631068

[5] Manthiram K Zhou Q Aksentijevich I Kastner DL. The monogenic autoinflammatory diseases define new pathways in human innate immunity and inflammation. Nat Immunol. (2017) 18:832–42. doi: 10.1038/ni.3777 28722725

[6] Ben-Chetrit E Gattorno M Gul A Kastner DL Lachmann HJ Touitou I. Consensus proposal for taxonomy and definition of the autoinflammatory diseases (AIDs): a Delphi study. Ann Rheum Dis. (2018) 77:1558–65. doi: 10.1136/annrheumdis-2017-212515 30100561

[7] Girardin SE Boneca IG Viala J Chamaillard M Labigne A Thomas G. Nod2 is a general sensor of peptidoglycan through muramyl dipeptide (MDP) detection. J Biol Chem. (2003) 278:8869–72. doi: 10.1074/jbc.C200651200 12527755

[8] Borzutzky A Fried A Chou J Bonilla FA Kim S Dedeoglu F. NOD2-associated diseases: Bridging innate immunity and autoinflammation. Clin Immunol. (2010) 134:251–61. doi: 10.1016/j.clim.2009.05.005 19467619

[9] Chen X Zhou Z Zhang Y Cheng X Guo X Yang X. NOD2/CARD15 gene polymorphisms and sarcoidosis susceptibility: review and meta-analysis. Sarcoidosis Vasc Diffuse Lung Dis. (2018) 35:115–22. doi: 10.36141/svdld.v35i2.6257 PMC7170086 32476890

[10] Yao Q Li E Shen B. Autoinflammatory disease with focus on NOD2-associated disease in the era of genomic medicine. Autoimmunity. (2019) 52:48–56. doi: 10.1080/08916934.2019.1613382 31084224

[11] Takada S Saito MK Kambe N. Blau Syndrome: NOD2-related systemic autoinflammatory granulomatosis. G Ital Dermatol Venereol. (2020) 155:537–41. doi: 10.23736/S0392-0488.19.06524-6 32618442

[12] Ashton JJ Seaby EG Beattie RM Ennis S. NOD2 in Crohn’s disease- unfinished business. J Crohns Colitis. (2022) 17:450–8. doi: 10.1093/ecco-jcc/jjac124 PMC10069614 36006803

[13] Yao Q Zhou L Cusumano P Bose N Piliang M Jayakar B. A new category of autoinflammatory disease associated with NOD2 gene mutations. Arthritis Res Ther. (2011) 13:R148. doi: 10.1186/ar3462 21914217 PMC3308076

[14] Yao Q Shen M McDonald C Lacbawan F Moran R Shen B. NOD2-associated autoinflammatory disease: a large cohort study. Rheumatol (Oxford). (2015) 54:1904–12. doi: 10.1093/rheumatology/kev207 26070941

[15] Yao Q Shen M Fernandez J. NOD2-associated autoinflammatory disease and immune deficiency. J Allergy Clin Immunol Pract. (2016) 4:780–2. doi: 10.1016/j.jaip.2016.02.016 27039238

[16] Yao Q Shen B. A systematic analysis of treatment and outcomes of NOD2-associated autoinflammatory disease. Am J Med. (2017) 130:365.e13–.e18. doi: 10.1016/j.amjmed.2016.09.028 27984003

[17] Yao Q Kontzias A. Expansion of phenotypic and genotypic spectrum in Yao syndrome: A case series. J Clin Rheumatol. (2022) 28:e156–e60. doi: 10.1097/RHU.0000000000001655 33394828

[18] Yao Q Su LC Tomecki KJ Zhou L Jayakar B Shen B. Dermatitis as a characteristic phenotype of a new autoinflammatory disease associated with NOD2 mutations. J Am Acad Dermatol. (2013) 68:624–31. doi: 10.1016/j.jaad.2012.09.025 23102769

[19] Yang X Wu D Li J Shen M Zhang W. A Chinese case series of Yao syndrome and literature review. Clin Rheumatol. (2018) 37:3449–54. doi: 10.1007/s10067-018-4274-0 30159790

[20] Ruzieh M Grubb BP. Orthostatic intolerance and postural tachycardia syndrome: new insights into pathophysiology and treatment. Herzschrittmacherther Elektrophysiol. (2018) 29:183–6. doi: 10.1007/s00399-018-0563-1 29696346

[21] Karamanakos A Vougiouka O Sapountzi E Venetsanopoulou AI Tektonidou MG Germenis AE. The expanding clinical spectrum of autoinflammatory diseases with NOD2 variants: a case series and literature review. Front Immunol. (2024) 15:1342668. doi: 10.3389/fimmu.2024.1342668 38348033 PMC10859468

[22] Shen M Moran R Tomecki KJ Yao Q. Granulomatous disease associated with NOD2 sequence variants and familial camptodactyly: An intermediate form of NOD2-associated diseases? Semin Arthritis Rheum. (2015) 45:357–60. doi: 10.1016/j.semarthrit.2015.05.007 26164256

[23] Yao Q Schils J. Distal lower extremity swelling as a prominent phenotype of NOD2-associated autoinflammatory disease. Rheumatol (Oxford). (2013) 52:2095–7. doi: 10.1093/rheumatology/ket143 23584365

[24] Yao Q Gorevic P Shen B Gibson G. Genetically transitional disease: a new concept in genomic medicine. Trends Genet. (2023) 39:98–108. doi: 10.1016/j.tig.2022.11.002 36564319

[25] Navetta-Modrov B Nomani H Yun M Yang J Salvemini J Aroniadis O. A novel nucleotide-binding oligomerization domain 2 genetic marker for Yao syndrome. J Am Acad Dermatol. (2023) 89:166–8. doi: 10.1016/j.jaad.2023.02.029 36858152

[26] Brailsford CJ Khamdan F Elston DM. Treatment of refractory Yao syndrome with canakinumab. JAAD Case Rep. (2022) 29:37–40. doi: 10.1016/j.jdcr.2022.08.035 36186408 PMC9522865

[27] Yao Q. Effectiveness of canakinumab for the treatment of patients with Yao syndrome. J Am Acad Dermatol. (2023) 88:653–4. doi: 10.1016/j.jaad.2019.09.020 31541750

