# Supplementary material for: Clinical phenotype, NOD2 genotypes, and treatment observations in Yao syndrome: a retrospective case series
Source: Front Immunol. 2024 Oct 4;15:1304792. doi: 10.3389/fimmu.2024.1304792 (PMC11486699; doi:10.3389/fimmu.2024.1304792)
Supplement: Supplementary Table 1 — Genetic Screening Methods for Autoinflammatory Disease. [file Table1.docx]

**Supplemental Table 1. Genetic Screening Methods for Autoinflammatory Disease.**

| Case Number | Genetic Testing |
| --- | --- |
| 1 | NOD2 Complete Gene Analysis (Center For Genetic Testing At St. Francis, Tulsa, OK)  GeneDX Custome Whole Exome Slice Autoinflammatory Gene Panel (116 genes) |
| 2 | NOD2 Complete Gene Analysis (Center For Genetic Testing At St. Francis, Tulsa, OK)  Invitae Primary Immunodeficiency Panel (474 genes) |
| 3 | NOD2 Complete Gene Analysis (Center For Genetic Testing At St. Francis, Tulsa, OK)  MEFV Full Gene Analysis  TNFRSF1A Full Gene Analysis |
| 4 | NOD2 Complete Gene Analysis (Center For Genetic Testing At St. Francis, Tulsa, OK)  Mayo Clinic PID Autoinflammatory Gene Panel (18 genes) |
| 5 | Invitae Autoinflammatory and Autoimmunity Syndromes Panel (156 genes) |
| 6 | NOD2 Complete Gene Analysis (Center For Genetic Testing At St. Francis, Tulsa, OK)  GeneDX Custome Whole Exome Slice Autoinflammatory Gene Panel (116 genes) |
| 7 | NOD2 Complete Gene Analysis (Center For Genetic Testing At St. Francis, Tulsa, OK)  GeneDX Custome Whole Exome Slice Autoinflammatory Gene Panel (116 genes) |
| 8 | AiLife Diagnostics Whole Exome Sequencing |
| 9 | NOD2 Complete Gene Analysis (Center For Genetic Testing At St. Francis, Tulsa, OK)  GeneDX Periodic Fever Syndromes Panel (7 genes) |
| 10 | NOD2 Complete Gene Analysis (Center For Genetic Testing At St. Francis, Tulsa, OK)  GeneDX Periodic Fever Syndromes Panel (7 genes) |
| 11 | NOD2 Complete Gene Analysis (Center For Genetic Testing At St. Francis, Tulsa, OK)  Invitae Primary Immunodeficiency Panel (474 genes)  GeneDX Whole Exome Sequencing |
| 12 | ARUP Periodic Fever Syndromes panel (10 genes) |
| 13 | NOD2 Complete Gene Analysis (Center For Genetic Testing At St. Francis, Tulsa, OK)  GeneDX Periodic Fever Syndromes Panel (7 genes) |
| 14 | NOD2 Complete Gene Analysis (Center For Genetic Testing At St. Francis, Tulsa, OK)  Variantyx Custome Whole Exome Slice Autoinflammatory Gene Panel (116 genes) |
| 15 | NOD2 Complete Gene Analysis (Center For Genetic Testing At St. Francis, Tulsa, OK)  GeneDX Periodic Fever Syndromes Panel (7 genes) |
| 16 | NOD2 Whole Gene Sequencing (Molecular Diagnostics Lab, Middlefield Ohio)  Invitae Autoinflammatory and Autoimmunity Syndromes Panel (156 genes) |
| 17 | NOD2 Complete Gene Analysis (Center For Genetic Testing At St. Francis, Tulsa, OK)  Invitae Primary Immunodeficiency Panel (474 genes)  GeneDX Whole Exome Reanalysis |
| 18 | Invitae Inborn Errors of Immunity and Cytopenias Panel (574 genes) |
| 19 | NOD2 Complete Gene Analysis (Center For Genetic Testing At St. Francis, Tulsa, OK)  Mayo Clinic PID Autoinflammatory Gene Panel (18 genes) |
| 20 | NOD2 Complete Gene Analysis (Center For Genetic Testing At St. Francis, Tulsa, OK)  Invitae Autoinflammatory and Autoimmunity Syndromes Panel (156 genes) |
| 21 | Invitae Autoinflammatory and Autoimmunity Syndromes and Primary Immunodeficiency Panel (429 genes) |
| 22 | Invitae Autoinflammatory and Autoimmunity Syndromes Panel (156 genes)  Invitae Connective Tissue Disorders Panel (245 genes) |
