# Supplementary material for: Clinical phenotype, NOD2 genotypes, and treatment observations in Yao syndrome: a retrospective case series
Source: Front Immunol. 2024 Oct 4;15:1304792. doi: 10.3389/fimmu.2024.1304792 (PMC11486699; doi:10.3389/fimmu.2024.1304792)
Supplement: Supplementary Table 2 — Laboratory results in the Mayo Clinic YAOS cohort. *Results show the number of patients with positive tests/number of patients (%) tested. †Immunofluorescence testing ranging from less than 1:40 to 1:320. Only one patient positive for ANA by immunofluorescence testing was found to have more specific autoantibodies (anti-SSB) and none demonstrated positive anti-double-stranded DNA antibodies. The second patient positive for anti-SSB had a negative ANA by immunofluorescence. ‡One patient was ANA negative. # One patient was PR3 positive with negative c-ANCA and p-ANCA immunofluorescence testing. [file Table2.docx]

**Supplemental Table 2. Laboratory results in the Mayo Clinic YAOS cohort.**

| Clinical Test | Results* |
| --- | --- |
| ANA | 7 / 22 (37) |
| Anti-dsDNA | 0 / 15 (0) |
| Anti-Smith | 0 / 17 (0) |
| Anti-RNP^‡^ | 1 / 17 (6) |
| Anti-SSA | 0 / 20 (0) |
| Anti-SSB*^†^* | 2 / 20 (11) |
| Anti-Scl70 | 0 / 13 (0) |
| Anti-Jo1 | 0 / 13 (0) |
| Anti-centromere | 0 / 5 (0) |
| Lupus anticoagulant | 0 / 7 (0) |
| Anticardiolipin antibodies | 0 / 10 (0) |
| Anti-beta-2-glycoprotein-1 antibodies | 1 / 8 (20) |
| Rheumatoid factor | 0 / 21 (5) |
| Anti-CCP antibodies | 0 / 18 (0) |
| ANCA*^#^* | 1 / 14 (7) |
| HLA-B27 | 0 / 11 (0) |

^*^Results show the number of patients with positive tests / number of patients (%) tested.

^†^Immunofluorescence testing ranging from less than 1:40 to 1:320. Only one patient positive for ANA by immunofluorescence testing was found to have more specific autoantibodies (anti-SSB) and none demonstrated positive anti-double-stranded DNA antibodies. The second patient positive for anti-SSB had a negative ANA by immunofluorescence .

^‡^One patient was ANA negative.

*^#^*One patient was PR3 positive with negative c-ANCA and p-ANCA immunofluorescence testing.
